# Reframing School Dropout as a Public Health Issue

**Published:** 2007-09-15

**Authors:** Nicholas Freudenberg, Jessica Ruglis

**Affiliations:** Hunter College School of Health Sciences, City University of New York; Graduate Center, City University of New York, New York, New York.

## Abstract

Good education predicts good health, and disparities in health and in educational achievement are closely linked. Despite these connections, public health professionals rarely make reducing the number of students who drop out of school a priority, although nearly one-third of all students in the United States and half of black, Latino, and American Indian students do not graduate from high school on time. In this article, we summarize knowledge on the health benefits of high school graduation and discuss the pathways by which graduating from high school contributes to good health. We examine strategies for reducing school dropout rates with a focus on interventions that improve school completion rates by improving students' health. Finally, we recommend actions health professionals can take to reframe the school dropout rate as a public health issue and to improve school completion rates in the United States.

## Introduction

If medical researchers were to discover an elixir that could increase life expectancy, reduce the burden of illness, delay the consequences of aging, decrease risky health behavior, and shrink disparities in health, we would celebrate such a remarkable discovery. Robust epidemiological evidence suggests that education is such an elixir. Yet health professionals have rarely identified improving school graduation rates as a major public health objective, nor have they systematically examined their role in achieving this objective. Seizing the opportunity to do so can improve health and reduce disparities.

## Impact of High School Graduation on Health

Education is one of the strongest predictors of health: the more schooling people have the better their health is likely to be. Although education is highly correlated with income and occupation, evidence suggests that education exerts the strongest influence on health (1-4). More formal education is consistently associated with lower death rates (4), while less education predicts earlier death. The less schooling people have, the higher their levels of risky health behaviors such as smoking, being overweight, or having a low level of physical activity (5). High school completion is a useful measure of educational attainment because its influence on health is well studied, and it is widely recognized as the minimum entry requirement for higher education and well-paid employment.

Although the beneficial effect of education varies by sex, age, and race/ethnicity, with blacks benefiting more than whites from more education (6), current policies exacerbate education-related health disparities, with women, whites, young adults, and United States–born residents having higher graduation rates than their respective counterparts (7). Moreover, the gap in health status between people who are well educated and those who are not has grown in recent decades (6).

## Pathways by Which Graduation Contributes to Improved Health

A good education leads to good health in several ways. First, the more schooling people have the more money they earn, enabling them to purchase better housing in safer neighborhoods, healthier food, better medical care and health insurance, and more education; each of these factors is associated with improved health (3,8,9). Each one allows individuals to move up the occupational and income ladder, giving them more prestige and power, both of which are associated with better health. High school completion is also the gateway into college, which offers even greater benefits than high school alone. Second, education facilitates healthier behavior choices by offering learners access to health information and tools to acquire help and resources such as smoking cessation programs. Third, education helps people to acquire social support, strengthen social networks, and mitigate social stressors (3,9,10). The more education people have the more social support they have (10). Education helps people to gain a sense of control over their lives (9), an outcome associated with better health.

According to a recent review by Cutler and Lleras-Muney (3), policies that increase educational attainment could have a large effect on population health. Moreover, estimates suggest that investments to improve educational achievement can save more lives than can medical advances (11). To realize these possibilities, public health researchers need to develop new conceptual and analytic approaches to studying the reciprocal relationships between health and education and consider education as an arena for intervention as well as a marker or moderator for social position (3,12).

## High School Graduation in the United States

In recent decades, educational attainment in the United States has improved significantly. From 1975 through 2000, the proportion of adults aged 25 years or older who completed high school increased from 63% to 84% (7). However, high dropout rates are increasingly concentrated among low-income and black and Latino students, and the rate at which students leave school between grades 9 and 10 has tripled (13). These trends indicate that more young adolescents are in jeopardy.

The Cumulative Promotion Index (CPI) (13) uses enrollment data to estimate the probability that a student entering 9th grade will graduate with a regular diploma in the traditional 4 years. Although many students finish high school in 5 or more years, the more narrowly defined CPI offers several advantages as a measure: it is commonly used, data are systematically collected, and it triggers the funding mandates set in the federal No Child Left Behind Act. The CPI method of calculating graduation shows that nearly one-third of students in the United States and half of black, Hispanic, and American Indian students who enter 9th grade do not graduate with a diploma in 4 years (Table 1).

Graduation rates in the nation's largest cities are lower still. In 2001, 6 of the 10 largest cities in the United States had overall graduation rates of less than 50% (Table 2). In 2002, 18% of the nation's 11,129 high schools promoted fewer than 60% of their students (15). Most of these schools with low promotion rates were concentrated in cities with low average incomes and with high proportions of blacks and Hispanics (15).

## Causes for School Dropout

Understanding why young people leave school can inform the design of polices that will increase school graduation rates. Although a comprehensive analysis of multidisciplinary studies of factors associated with school completion is beyond the scope of this article, Table 3 summarizes findings from social science and educational research on dropout rates, assessing the impact of factors from different levels of society (e.g., individual, community, school). The multiple factors associated with dropout rates suggest that no single type of intervention can end our nation's dropout crisis.

Although much of the research on school completion focuses on the psychological traits of students and the organizational characteristics of teachers, schools, and school systems, some researchers have examined the impact of health. Health has direct and indirect effects on school dropout rates. Student health problems associated with dropping out are substance use; pregnancy; and psychological, emotional, and behavioral problems (27-30). Teenage pregnancy is the leading cause of dropping out of school for adolescent women; an estimated 30%–40% of female teenaged dropouts are mothers (29). Early parenting also affects young men who drop out to support a child.

Mental illness and emotional disturbance also account for a significant percentage of dropouts (31). Health problems also affect dropout rates indirectly by forcing young people, especially young women, to cope with family physical or mental illness, often imposing on teenagers responsibilities that can lead to their leaving school (32). The few researchers who examined the impact of addiction, mental illness, chronic diseases, or mortality among parents on students' school achievement suggest it has a substantial effect (33,34).

## Health Interventions

Interventions to reduce school dropout rates seek to change individuals, families, schools, school systems, or public policies related to poverty, welfare, or employment. Most educational research has focused on evaluating interventions designed to alter the school curriculum, improve support for teachers, or change the institutional mindset in schools, as summarized in Table 4.

Interventions that have the potential to improve school achievement and reduce school dropout rates by improving the health of students are of particular interest to health professionals. These school-based interventions include coordinated school health programs; health clinics; mental health programs; substance abuse prevention and treatment programs; comprehensive sex education, human immunodeficiency virus infection prevention, and pregnancy prevention programs; special services for pregnant and parenting teens; violence prevention programs; and interventions to change the schools' social climate (29,31,43-49). Table 5 lists the approaches that have the potential to reduce dropout rates. Although the focus here is on adolescents, these approaches are also used in elementary and middle schools. In addition, community-based programs can also promote adolescent health but are beyond the scope of this article.

Many schools offer several different types of health programs shown in Table 5. However, these activities are seldom coordinated, and they do not target reducing school dropout rates as an outcome. Few innovative or effective programs have gone beyond pilot studies or have been provided funding that assured sustainability. Evaluation studies that assess the impact of health programs on school dropout rates are rare, a disturbing gap given the importance of school dropout as a health, social justice, and economic issue. As a result, a comprehensive framework explaining the mechanisms by which various types of health programs reduce dropout rates is not available, making it difficult for school or health officials to select the most effective interventions for their setting.

## Recommendations

Although evidence shows that education is an important determinant of health and that changes in school policy can improve educational outcomes, public health professionals have seldom made improving school completion rates a health priority. In addition, poor health interferes with children's capacity for education, and a variety of school-based health interventions have the potential to improve school achievement. With a few important exceptions, health providers have not developed lasting partnerships with schools, nor have researchers provided the evidence needed to improve or replicate health programs that can reduce school dropout rates.

Improving graduation rates is a specific objective that can bring health professionals and educators together for research, intervention, and advocacy to improve the lives and well-being of young people. We suggest five priorities for action. Local implementation will, of course, depend on which constituencies are mobilized, but every community can take some action to make the link between health and school completion a priority for action.

1. Target schools and cities with the most serious dropout problems for intensive intervention. In the United States, about 1000 high schools fail to graduate half their students, and in more than 20 cities at least three-quarters of high school students attend schools where fewer than 60% of students graduate (14). These appalling statistics undermine health, economic development, and social justice, and they serve as powerful generators of disparities in health. To reduce school dropout rates, the National Research Council Panel on High-Risk Youth recommended in 1993 that "the primary institutions that serve youth — health, schools, employment, training — are crucial and we must begin with helping them respond more effectively to contemporary adolescent needs. Effective responses will involve pushing the boundaries of these systems, encouraging collaborations between them and reducing the number of adolescents whose specialized problems cannot be met through primary institutions" (59, p. 193). A good first step would be to create state or municipal intersectoral dropout prevention councils in places where there is a disproportionate number of dropouts. Such councils could design, seek funding for, implement, and evaluate the educational, vocational, antipoverty, and health interventions at the intensity and scale needed to improve school completion rates in their areas.
2. Develop, implement, and evaluate health interventions to improve school completion rates. The paucity of research that explores the reciprocal connections between health and school achievement makes the development of a coordinated research agenda that will better identify health-related determinants of children dropping out of school an urgent priority. Such an agenda could guide the selection and evaluation of interventions to reduce dropout rates. Two promising avenues for research are studies of health interventions that better engage young people in their schools and that connect young people to caring adults. Schools that foster student engagement in their studies are more likely to graduate their students (35,60), and young people who feel connected to at least one adult in their school are much more likely to graduate (35). Some intervention research suggests that changes in school climate can increase students' connection to adults and their level of engagement in their studies (58). Health interventions, including those targeted at sexual and reproductive health, healthy relationships, family health, violence prevention, substance use, and mental health, have the potential to engage young people in schooling and connect them to caring adults.
3. Strengthen support for health education teachers. Developing and implementing new approaches to school-based health education and health services that can reduce dropout rates will require well-trained school health education teachers, nurses, and mental health professionals, each currently in short supply. Better integration between health education and services in the school and community, consistent funding for school health education, partnerships between schools and universities, and strong professional preparation programs for health education teachers can help to reduce dropout rates by addressing student, family, and community health.
4. Advocate for evidence-based interventions that can improve health and reduce dropout rates. Health professionals can play a positive role in the contentious debates about providing services in schools addressing sex education; substance abuse; birth control, pregnancy, and parenting services; violence prevention; and mental health. By bringing evidence of effectiveness and public support into public deliberations on these issues, offering science-based arguments in support of interventions addressing these issues, joining coalitions that can compete effectively in the political arena, and explaining the links between health and education, health professionals can contribute to more informed public participation.
5. Put reducing high school dropout rates on the public health agenda. The public health community can bring its expertise in advocacy to the campaign to make improving graduation rates a high national priority. Simply reframing school dropout as a health issue has the potential to bring new players into the effort — parents, health institutions, young people, civil rights groups — and to encourage public officials to think of the dropout problem as central to community health and as a long-term solution beneficial to population health. Educating the public and policy makers about the long-term benefits of improved school completion (e.g., reductions in socioeconomic and racial/ethnic health disparities, lifetime health care costs, unhealthy behavior) can provide additional incentives for action. More specifically, public health professionals can advocate for good school health programs and can encourage administrators of these programs to make improving school completion a key objective. As citizens, taxpayers, parents, and advocates for social justice, public health professionals can join the fight for equitable funding and staffing of schools as well as advocating for school systems to be rated on their success in improving school completion through fair and equitable means.

## Conclusion

Seldom have health and education professionals been in a better position to work together to achieve common goals. Rarely has a single problem — high school dropout rates — contributed to so many adverse social, economic, and health conditions. Our nation's young people deserve no less than a concerted effort to improve school completion rates and thus give young people a gateway to lifetime health and success.

It is not possible to eliminate health disparities without simultaneously reducing disparities in educational achievement. The populations that are most severely affected by the epidemics that have threatened this nation's health in the last several decades are the populations most at risk of dropping out of school. By bringing together programs to improve health and school achievement and by making reducing school dropout rates a public health, educational, and human rights priority, public health professionals have the opportunity to make a lasting contribution to promoting population health and social justice.

## Figures and Tables

**Table 1:** National Graduation Rates, by Race or Ethnicity and Sex, United States, 2001

Race or Ethnicity	Female %	Male %	Total %
American Indian/Alaska Native	51.4^a^	47.0^a^	51.1
Asian/Pacific Islander	80.0^a^	72.6^a^	76.8
Black	56.2	42.8	50.2
Hispanic	58.5	48.0	53.2
White	77.0	70.8	74.9
**All students**	**72.0**	**64.1**	**68.0**

Source: Swanson CB (14).

a Rate based on estimates that cover between 50% and 75% of the student population.

**Table 2:** Graduation Rates for the 10 Largest Public School Districts in the United States, 2001

**District (Enroll-ment)**	Characteristic			Cumulative Promotion IndexGraduation Rates, %					

	Largest Racial or Ethnic Group	% Min-ority^a^	% Free or Re-duced Lunch^b^	Total	American Indian	Asian	Hispanic	Black	White
New York City, NY (1,066,516)	Hispanic	84.7	71.9	38.2	41.2	60.9	30.1	32.2	57.9
Los Angeles Unified School District, CA (721,346)	Hispanic	90.1	73.5	46.4	50.8	76.6	40.2	48.1	68.1
City of Chicago, IL (435,261)	Black	90.4	—	48.4	—	80.6	50.8	42.1	65.3
Dade County, FL (368,625)	Hispanic	88.7	59.3	52.1	—	84.7	52.8	46.8	60.7
Broward County, FL (251,129)	White	58.8	37.1	47.2	49.5	79.5	—	35.2	55.7
Clark County, NV (231,655)	White	50.1	26.3	51.9	51.5	79.1	37.3	40.1	58.7
Houston Indepen-dent School District, TX (208,462)	Hispanic	90.0	70.7	40.2	—	78.1	34.7	39.5	62.3
Philadelphia City, PA (201,190)	Black	83.3	66.7	41.9	27.1	59.5	31.5	41.1	45.6
Hawaii Depart-ment of Education, HI (184,360)	Asian	79.6	43.7	66.0	70.9	66.8	59.9	60.7	64.7
Hills-borough County, FL (164,311)	White	48.2	47.4	55.0	—	86.3	51.0	41.5	60.2

Dashes (—) indicate that district provided no data for this group. Source: Swanson CB (14).

a Indicates percentage of nonwhite students enrolled in the district.

b Indicates percentage of students in the district eligible for federal free or reduced-cost lunch programs, a proxy for poverty and socioeconomic status.

**Table 3:** Summary of Factors Associated With Dropping Out of School

Individual or Family	Neighborhood or Community	School or School System
Low family socioeconomic status Racial or ethnic group Male Special education status Low family support for education, less opportunity for nonschool learning, few study aids and resources in the home Low parental educational attainment Residential mobility Low social conformity Low acceptance of adult authority High levels of social isolation Behaviors such as disruptive conduct, truancy, absenteeism, and lateness Being held back in school Poor academic achievement, low grades or test scores Academic problems in early grades Not liking school Feelings of "not fitting in" and of not belonging Perceptions of unfair or harsh disciplines Feeling unsafe in school Not engaged in school Being suspended or expelled Conflicts between work and school Having to work or support family Substance use Pregnancy	Living in a low-income neighborhood Having peers with low educational aspirations Having friends or siblings who are dropouts	Low socioeconomic status of school population High level of racial or ethnic segregation of students between schools in a district or within tracks or classes in a building High proportion of students of color in school High proportion of students enrolled in special education Location in central city Large school district School safety and disciplinary policies High-stakes testing High student-to-teacher ratios Academic tracking Discrepancy between the racial or ethnic composition of students and faculty Lack of programs and support for transition into high school for 9th and 10th graders
References: 16-20	References: 21-23	References: 16, 24-26

**Table 4:** Summary of Educational Interventions for Improving Student Engagement in School and Academic Success

Structural, Institutional, and Organizational Changes	Changes to Curriculum and Instruction	Changes in Teacher Support
Safe, nonthreatening learning environment Small class size Small school size Systemic, comprehensive school reform Culturally proficient leadership Community, business, and university collaboration Student involvement in school policies Reducing retention and suspension Efforts focused on 9th grade transition Small learning communities Parent and family training and involvement Violence prevention and conflict resolution programs Culturally competent school and classroom culture Alternative school safety and fair discipline strategies Alternative school models: school-to-work programs, apprenticeship, vocational, service learning	Extend class periods or increase instructional time Opportunities for "catch up" courses and for out-of-school programs Academic content that is of interest and relevance to the students Academic and social supports for students Advisory periods Elimination of academic tracking Student-centered, culturally relevant, and diverse pedagogy and practice Opportunities for extra schooling: after school, summer, Saturday, or extended-day school Fair, clear, rigorous, and high expectations and standards for all students Tutoring Mentoring programs Behavioral and psychosocial support Efforts to build relationships, foster school engagement and social support, and reduce alienation Diverse and individualized instruction and use of instructional technologies Early intervention and academic supports Interdisciplinary instruction	Common planning times Integrated interdisciplinary planning processes Professional development Coaching and mentoring Comprehensive teacher training Support for staff risk-taking, self-governance, and collaboration Collective responsibility and increased autonomy from central control Highly qualified, certified, and well-prepared teachers Teachers teaching only in their field of certification Education programs to help teachers promote social justice Teacher training for effective instruction of and care for culturally and linguistically diverse learners
Sources: 19, 35–42	Sources: 19, 35–38, 40	Sources: 19, 38, 40, 41

**Table 5:** Health Interventions That May Contribute to Improved School Completion Rates

Type of Intervention (Selected References)	Program Activities	How the Intervention Reduces Dropout Rates
Coordinated school health program (43,50)	Health education; physical education; health services; nutrition services; counseling, psychological, and social services; healthy school environment; health promotion for the staff, family, and community; partnerships	Teaches decision-making skills for better life choices; reduces absenteeism; offers early intervention and referrals for learning, psychological, substance abuse, and mental health problems; makes school more engaging; connects students to caring adults; engages families and communities in lives of young people
School-based health clinic (51,52)	Primary and preventive health care, referrals, assistance in finding health insurance and health care for family, reproductive health services, mental health counseling	Reduces family health problems; offers early intervention and treatment for psychological and physical health problems that can interrupt schooling; reduces teen pregnancy
Mental health programs (31,53)	Assessment and early intervention for young people with psychological, learning, or behavioral problems; referrals for children and families; counseling; staff training	Prevents problems that can interfere with school from becoming more serious; connects young people to caring adults; makes school more engaging; provides counseling or referrals for family mental health problems
Substance abuse prevention and treatment programs (45,54)	Alcohol, tobacco, and drug use prevention education; peer education; early intervention for drug users; support for young people with substance-abusing parents; referrals for drug treatment or counseling	Reduces or delays onset of heavy alcohol or marijuana use; offers young people with a drug-using parent a source of support; makes school more engaging
Sex, HIV infection, and pregnancy prevention programs (46,47,55)	Sex education; HIV infection prevention services; referrals for reproductive and sex health services; birth control; peer education; sexually transmitted infection prevention	Reduces or delays teen pregnancy; connects young people to caring adults or peers who encourage healthy behavior
Services for pregnant and parenting teens (29,56)	Child care; parenting education; reproductive health services; continued participation in high school academics/courses	Encourages and supports teen mothers to continue schooling; delays second pregnancy
Violence prevention programs (47,57)	Peer education/mediation; anger management; conflict resolution; violence prevention education; psychosocial services; individual and group counseling	Makes young people feel safer in school; makes school more engaging; connects young people to caring adults or peers who encourage healthy behavior
School climate (49,58)	Policy changes to reduce stigmatization, bullying, aggressive policing, or punitive disciplinary measures; peer education; increased opportunities for close adult-student interactions	Improves student engagement in school activities; connects young people to caring adults; reduces bullying, stigmatization, and distrust of authority

HIV indicates human immunodeficiency virus.
